# The Central Role of the Global Emerging Infections Surveillance Program in Supporting Force Health Protection 

**DOI:** 10.3201/eid3014.240304

**Published:** 2024-11

**Authors:** June M. Early, Hunter J. Smith, Stephanie S. Cinkovich, M. Shayne Gallaway, Christan N. Stager, Matthew R. Kasper

**Affiliations:** Global Emerging Infections Surveillance (GEIS) Branch, Armed Forces Health Surveillance Division, Defense Health Agency, Silver Spring, Maryland, USA (J.M. Early, H.J. Smith, S.S. Cinkovich, M.S. Gallaway, C.N. Stager, M.R. Kasper); Cherokee Nation Businesses, Tulsa, Oklahoma, USA (S.S. Cinkovich)

**Keywords:** Global Emerging Infections Surveillance program, GEIS, infectious disease, surveillance, military

## Abstract

The Global Emerging Infections Surveillance (GEIS) program is the only Department of Defense (DoD) organization that coordinates global surveillance for emerging infectious diseases that affect US military forces operating in the United States or foreign locations. Since 1997, the GEIS program has focused on surveilling pathogens likely to affect military operations and the health of service members. The foundation of the GEIS program is the long-standing, mutually beneficial relationships between the DoD overseas laboratories and their host-country partners and militaries. Through centralized programmatic support, the GEIS program provides the infrastructure needed for a rapid and scalable response to emerging threats. The GEIS program continues to enhance and evolve its initiatives to provide timely, reliable information to decision-makers in the DoD. The GEIS program has been and will continue to be a vital source of actionable biosurveillance information during infectious disease events of global public health concern.

In 1995, subject matter experts and policymakers in the US government convened to discuss the global threat of infectious diseases (*1*). With increasing urbanization, global interconnectedness, antimicrobial drug resistance, and climate change, experts called for increased capabilities and capacity to surveil for infectious diseases globally. To answer this call, in 1996, the Clinton Administration issued Presidential Decision Directive—National Science and Technology Council (NSTC)-7 on emerging infectious diseases. The NSTC-7 tasked the Department of Defense (DoD) to expand its mission to include support of global surveillance, training, research, and response to emerging infectious disease threats (*2*). NSTC-7 further charged the DoD to strengthen centralized coordination and epidemiologic capabilities to control and reduce disease. In response to that directive, the Assistant Secretary of Defense for Health Affairs established the DoD’s Global Emerging Infections Surveillance (GEIS) program in 1997 with the primary mission of protecting US military forces from infectious disease threats at home and abroad (*3*). The renewed concern and interest in emerging pathogenic threats proved to be warranted, as the 21st century would bring multiple devastating emerging infectious disease events, including pandemic H1N1 (*4*), severe acute respiratory syndrome coronavirus (*5*), Middle East respiratory syndrome coronavirus (*6*), novel antimicrobial-resistant threats (*7*), Ebola virus (*8*), Zika virus (*9*), and more recently, SARS-CoV-2 (*10*). In this article, we provide context for how the GEIS program was initiated and has evolved, including strengths of the GEIS partner network and current priorities for infectious disease surveillance.

Upon establishment, the GEIS program was designated as the central hub for DoD infectious disease surveillance efforts. Over time, the program grew in line with US government funding for surveillance of emerging infectious disease threats, including multiple public health events of international concern (e.g., highly pathogenic avian influenza H5N1, pandemic H1N1, Ebola virus, etc.). The GEIS program has been part of the DoD’s Armed Forces Health Surveillance Division since 2008 (*11*) and the Defense Health Agency’s (DHA) Public Health Directorate since 2015.

The GEIS program, through its network partners, conducts surveillance for military-relevant infectious diseases in service members or proxy populations where the US military operates; forges and maintains collaborative relationships with partner militaries and host nations to advance health diplomacy and strengthen global health security; sustains infrastructure, expertise, and technology needed to detect and characterize emerging or concerning infectious disease threats to promote readiness; and enables a coordinated network of laboratories in austere locations overseas and more technically advanced reach-back laboratories in the United States. Data and information outputs from those efforts are curated for the unique needs of diverse DoD decision-makers.

Historically, GEIS funding has been directed to all 3 branches of the armed forces: Army, Navy, and Air Force, and more recently, the Tri-Service DHA. However, GEIS has no direct command authority over the laboratories or organizations it funds. Most GEIS funding is distributed to the 6 DoD overseas laboratories: Walter Reed Army Institute of Research (WRAIR)-Armed Forces Research Institute of Medical Science (Bangkok, Thailand), Naval Medical Research Unit (NAMRU) EURAFCENT (headquartered in Sigonella, Italy, with detachments in Ghana and Egypt), NAMRU INDO PACIFIC (Singapore), NAMRU SOUTH (Lima, Peru), WRAIR-Africa (Nairobi, Kenya), and WRAIR-Europe and Middle East (Tbilisi, Republic of Georgia).

Those overseas laboratories, in close collaboration with their host-country partners, serve as forward sites for collection of specimens (e.g., human, animal, environmental); vectors (e.g., mosquitoes, ticks); isolates (e.g., bacterial, viral); and other relevant epidemiologic data for further advanced characterization (e.g., whole-genome sequencing) and analyses. In addition, funding is provided for surveillance programs across military installations and the Military Health System, in the United States and abroad, to monitor military-relevant infectious diseases, such as seasonal influenza and multidrug-resistant organisms. The GEIS program also routinely coordinates with other entities within the DoD, US government agencies, and international organizations to rapidly communicate information in response to infectious disease threats.

## Scope and Value of the GEIS Network

The GEIS program’s funded activities are organized into 3 focus areas: antimicrobial-resistant infections, febrile and vectorborne infections, and respiratory infections. All GEIS-funded surveillance activities across those focus areas center strictly around infectious diseases that are relevant to force health protection (i.e., relevant to military health in support of operational readiness). The antimicrobial resistant infections focus area surveils for multidrug-resistant organisms detected in nosocomial infections, community-acquired infections, and wound and trauma settings; sexually transmitted infections; and enteric infections. The febrile and vectorborne infections focus area provides surveillance and support for causes of undifferentiated acute febrile illness; vectorborne and zoonotic pathogens, vectors, and reservoir hosts of relevant infections; insecticide resistance; and the effectiveness of malaria countermeasures (e.g., antimalarials, rapid diagnostic tests). The respiratory infections focus area surveils for known and unknown respiratory pathogens and supports studies of vaccine effectiveness and potential shift and drift within influenza subtypes that might be associated with increased severity and transmission of respiratory infections.

The DoD overseas laboratories, many of which have been in existence for decades, maintain critical, long-standing relationships with allied militaries and partner nations, enabling collaborative detection and reporting of known, novel, and emerging infectious disease threats and bidirectional information exchange. To complement capabilities of the overseas laboratories, the GEIS program funds US-based reach-back laboratories for next-generation sequencing, bioinformatics, external quality assurance, species confirmation, and other supporting functions. The network connections among the US-based DoD laboratories, the overseas laboratories, and other partners contribute to the global footprint of the GEIS program (Figure) and ensure that the DoD can quickly detect, understand, and respond to emerging threats as they arise anywhere around the world where service members might be located. The geographic reach of the GEIS program enables an adaptable response to evolving threats wherever they may emerge worldwide, provides a robust source of biosurveillance data and information, and leads to more relevant insights for decision-makers, better protecting the health and readiness of our armed forces.

**Figure:**
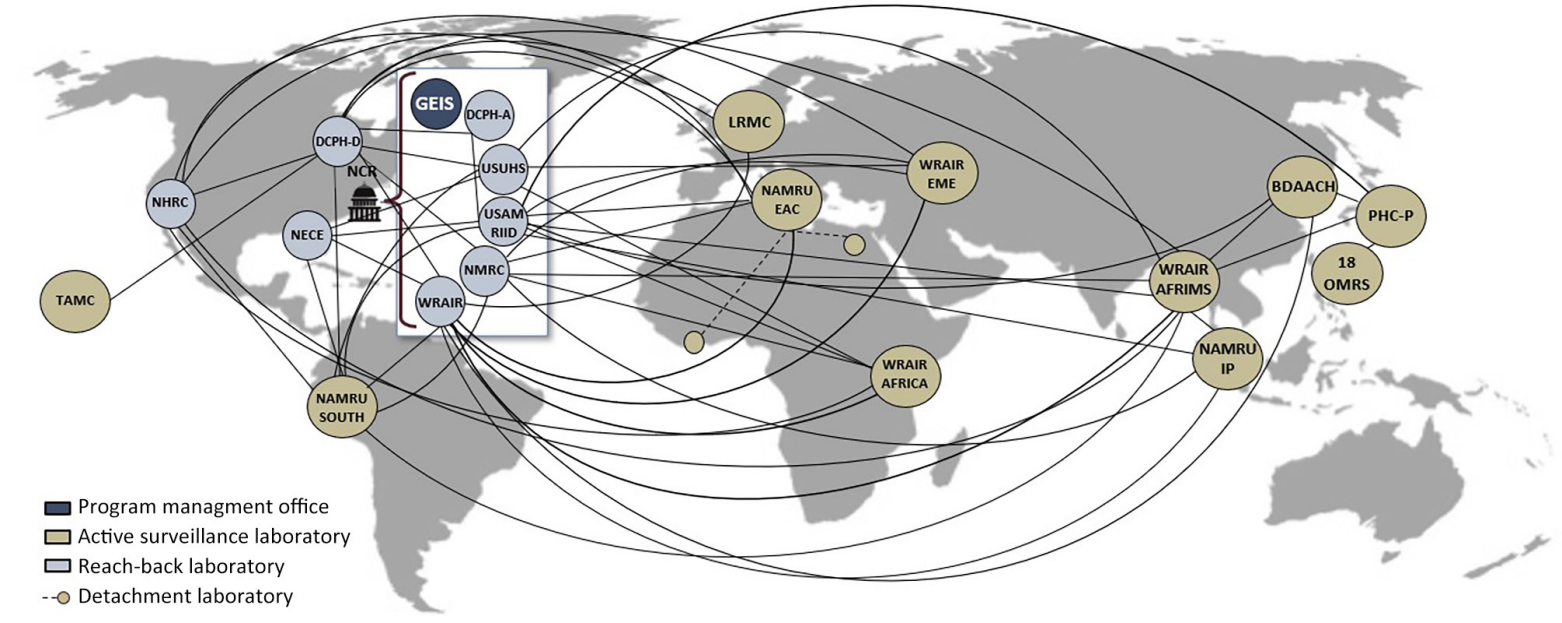
Interconnectivity of laboratories across the GEIS Program network. 18 OMRS, 18 Operational Medical Readiness Squadron (Okinawa, Japan); BDAACH, Brian D. Allgood Army Community Hospital (Pyeongtaek, South Korea); DCPH-A, Defense Center for Public Health—Aberdeen (Aberdeen, Maryland, USA); DCPH-D, Defense Center for Public Health—Dayton (Dayton, Ohio, USA); GEIS, Global Emerging Infections Surveillance (Silver Spring, Maryland, USA); LRMC, Landstuhl Regional Medical Center (Landstuhl, Germany); NAMRU EAC, Naval Medical Research Unit EURAFCENT (Sigonella, Italy); NAMRU IP, Naval Medical Research Unit Indo Pacific (Singapore); NAMRU SOUTH, Naval Medical Research Unit South (Lima, Peru); NCR, National Capital Region; NECE, Navy Entomology Center of Excellence (Jacksonville, Florida, USA); NHRC, Naval Health Research Center (San Diego, California, USA); NMRC, Naval Medical Research Command (Silver Spring, Maryland, USA); PHC-P, Public Health Command—Pacific (Okinawa, Japan); TAMC, Tripler Army Medical Center (Honolulu, Hawaii, USA); USAMRIID, US Army Medical Research Institute of Infectious Diseases (Fredrick, Maryland, USA); USUHS, Uniformed Services University of the Health Sciences (Bethesda, Maryland, USA); WRAIR, Walter Reed Army Institute of Research (Silver Spring, Maryland, USA); WRAIR AFRICA, Walter Reed Army Institute of Research Africa (Kisumu, Kenya); WRAIR AFRIMS, Walter Reed Army Institute of Research—Armed Forces Research Institute of Medical Science (Bangkok, Thailand); WRAIR EME, Walter Reed Army Institute of Research Europe-Middle East (Tbilisi, Georgia).

The GEIS program has formed and maintained flexible and scalable capabilities (e.g., personnel, equipment, and the supplies and reagents needed for sample collection and basic pathogen identification) for pandemic preparedness and response with guidance and coordination through its role as a central hub. An example of this centralized network coordination is GEIS’ Next Generation Sequencing and Bioinformatics Consortium (*12*), which is composed of subject matter experts from DoD laboratories around the world working to promote standardization and best practices, ultimately increasing the quality and utility of microbial genomic surveillance data. This high-quality and timely genomic surveillance data enables earlier detection and more rapid communication of novel and emerging infectious disease threats, enabling earlier interventions to support force health protection.

The long-standing regional partnerships between DoD laboratories and host-nation collaborators, combined with decades-long investment from the GEIS program, have advanced health diplomacy and global health security. Support from the GEIS program enables the surveillance of high-consequence pathogens and the collection of associated data, which are shared with local countries for public health decision-making. The partnerships between GEIS-supported DoD laboratories and local militaries and Ministries of Defense are also a unique tool for maintaining strong alliances with partner nations and strengthening global health security. Local surveillance capabilities and laboratory expertise have been built and sustained through GEIS-funded activities that are conducted by host-nation scientists and professionals in the partner countries where surveillance is conducted. This ensures expertise and infrastructure exist so that resources are in place to respond rapidly when an emerging threat is detected.

Data and downstream products routinely generated by the GEIS program are distributed to a multidisciplinary audience of decision-makers, clinicians, infection control preventionists, veterinarians, and other public health authorities. GEIS staff and subject matter experts ensure that data generated from surveillance activities are contextualized and capable of contributing to harmonized, military-relevant guidance. The infectious disease surveillance activities and subsequent reports and informational products result in data and information used to inform routine surveillance, countermeasure development, infection-control practices, force health protection posture, public health policy, outbreak detection and investigation, clinical practice guidelines, and more (Table).

**Table:** Select accomplishments of the Global Emerging Infections Surveillance Program in supporting force health protection, 1997–2024*

No.	Surveillance priority	Accomplishment
1	Influenza	Data on circulating influenza strains collected from GEIS-funded partners from ≈400 locations in >30 countries around the world directly informs the selection of strains in the annual influenza vaccination, leading to a safe and effective preventive countermeasure for service members and civilians alike.
2	Multidrug-resistant organisms	With funding support from the GEIS program, the WRAIR MRSN used genomic data from whole-genome sequencing to detect an outbreak of *Pseudomonas aeruginosa* in a military treatment facility, ultimately leading to the identification of an environmental reservoir as the source of transmission (W. Stribling et al., unpub. data, https://www.biorxiv.org/content/10.1101/2023.07.24.550326v2).
3	*Plasmodium falciparum* malaria	Data on *pfhrp2/3*-deleted *P. falciparum* parasites collected from GEIS-funded partners in Africa, Asia, and South America demonstrated that rapid diagnostic tests, like the DoD-authorized BinaxNOW, might not be suitable for accurately diagnosing malaria in countries where service members are or could be deployed, highlighting the need for the DoD to seek alternative diagnostics (*13*).
4	SARS-CoV-2 variants of concern	With funding support from the GEIS program, DoD laboratories were able to rapidly detect emerging SARS-CoV-2 variants of concern, including Alpha (N501Y) in Kenya (*14*), and Omicron (B.1.1.529) and Delta (B.1.617.2) in the United States.
5	Acute diarrhea	The GEIS program coordinated with DoD subject matter experts and decision-makers to develop clinical practice guidelines for management of acute diarrhea, a common medical condition with a significant operational impact, in the Middle East.

*DoD, Department of Defense; GEIS, Global Emerging Infections Surveillance; WRAIR MSRN, Walter Reed Army Institute of Research Multidrug-Resistant Organism Repository and Surveillance Network.

As part of its coordinating role, the GEIS program also shares surveillance data generated from funded projects to inform DoD’s medical research and development pipeline (e.g., therapeutics, vaccines, diagnostics, etc.). Although the GEIS program does not provide funding for conducting research for countermeasure development or testing and evaluating diagnostics, it does maintain active surveillance sites and laboratory testing capabilities through funding to DoD overseas laboratories and other DoD sites. Those sites generate data and pathogen isolates that are stored in repositories that can later be used to evaluate medical countermeasures and diagnostics. The GEIS program also coordinates closely with the Centers for Disease Control and Prevention and others through information sharing and programmatic reviews. Activities and portfolios across those programs are shared for visibility, opportunities for collaboration, and to reduce redundancy.

## Current Strategic Vision and Looking Ahead

Recognizing that humans, animals, and our environment are all interconnected, the GEIS program conducts surveillance across One Health domains (*15*), leading to a more comprehensive understanding of how infectious diseases emerge and are transmitted. In addition, the GEIS network performs pathogen-agnostic sequencing (*16*) when the causative agent of a disease is unknown or when a genome has not been previously characterized. GEIS also conducts wastewater surveillance (*17*) as a tool to detect pathogens circulating in a given location. The GEIS program is seeking to leverage its One Health, pathogen-agnostic sequencing, and wastewater surveillance capabilities to support biodefense and biosecurity efforts aimed at preventing the next pandemic (*18*).

The GEIS program recognizes the growing importance of data stewardship and modernization and is invested in technology solutions to streamline management of surveillance and programmatic data. The GEIS program has begun an initiative to deploy a centralized health information system for collection, management, analysis, and reporting of standardized data from the DoD overseas laboratories. Data collected in the field and input into the health information system would then feed into a central, DHA-hosted data lake for additional access, curation, and analysis by DoD and GEIS personnel. Lowering the manual entry, analytic, and reporting burdens on GEIS partners will lead to a more efficient use of GEIS program funds and improve the transparency of the results of GEIS activities. In addition, to overcome the challenges of sharing, storing, and analyzing genomic data, the GEIS program is partnering with other organizations in the DoD to implement a secure, accessible, cloud-based solution for storing and analyzing genomic data submitted by partners across the globe. The GEIS program continues to refine its approach to data collection, storage, analysis, transfer, and reporting to improve communication and improve early warning of emerging threats for informed decision-making.

## Conclusions

Since 1997, the GEIS program has been the only DoD organization coordinating global surveillance for emerging infectious disease threats, focusing on those likely to affect military operations and the health and readiness of service members. The emergence and spread of pandemic-level pathogens has illustrated the need for continued, well-resourced programs focused on detecting those threats and providing early warning for public health decision-making. The GEIS program has built, supported, and maintained a coordinated, responsive network of geographically diverse laboratory capabilities that can quickly pivot in response to an emerging threat. The GEIS program continues to evolve and expand its approach to surveillance by implementing pathogen-agnostic sequencing, wastewater surveillance, and increasing the value of traditional disease surveillance activities. The success of the GEIS program is due in large part to the long-standing partnerships, relationships, and agreements between the DoD overseas laboratories and their host countries. Those continued long-term efforts enhance larger health diplomacy efforts between the United States and ally nations abroad by supporting collaborations on infectious pathogens of relevance for both the host nations and the US military. The expertise the GEIS program has cultivated has become a vital tool within the DoD and US government for expeditiously and comprehensively identifying, characterizing, and reporting data on military-relevant pathogens that threaten the health and readiness of US forces.

## References

[1] Infectious disease—a global health threat: report of the National Science and Technology Council, Committee on International Science, Engineering, and Technology, Working Group on Emerging and Re-emerging Infectious Diseases. Washington, DC: Executive Office of the President of the United States; 1995.

[2] The White House Office of Science and Technology Policy. Fact sheet addressing the threat of emerging infectious diseases. June 12, 1996.

[3] Institute of Medicine Committee to Review the Department of Defense Global Emerging Infections Surveillance and Response System. In: Brachman PS, O’Maonaigh HC, Miller RN, editors. Perspectives on the Department of Defense Global Emerging Infections Surveillance and Response System: a program review. Washington, DC: National Academies Press; 2001. p. 1-29. 25057577

[4] Dawood FS, Jain S, Finelli L, Shaw MW, Lindstrom S, Garten RJ, et al.; Novel Swine-Origin Influenza A (H1N1) Virus Investigation Team. Emergence of a novel swine-origin influenza A (H1N1) virus in humans. N Engl J Med. 2009;360:2605–15. 10.1056/NEJMoa0903810 19423869

[5] World Health Organization. Report of the WHO–China Joint Mission on Coronavirus Disease 2019 (COVID-19) [cited 2024 Jan 10]. https://www.who.int/publications/i/item/report-of-the-who-china-joint-mission-on-coronavirus-disease-2019-(covid-19)

[6] Omrani AS, Al-Tawfiq JA, Memish ZA. Middle East respiratory syndrome coronavirus (MERS-CoV): animal to human interaction. Pathog Glob Health. 2015;109:354–62. 10.1080/20477724.2015.1122852 26924345 PMC4809235

[7] Michael CA, Dominey-Howes D, Labbate M. The antimicrobial resistance crisis: causes, consequences, and management. Front Public Health. 2014;2:145. 10.3389/fpubh.2014.00145 25279369 PMC4165128

[8] Bell BP, Damon IK, Jernigan DB, Kenyon TA, Nichol ST, O’Connor JP, et al. Overview, control strategies, and lessons learned in the CDC response to the 2014–2016 Ebola epidemic. MMWR Suppl. 2016;65:4–11. 10.15585/mmwr.su6503a2 27389903

[9] Chang C, Ortiz K, Ansari A, Gershwin ME. The Zika outbreak of the 21st century. J Autoimmun. 2016;68:1–13. 10.1016/j.jaut.2016.02.006 26925496 PMC7127657

[10] Hu B, Guo H, Zhou P, Shi Z-L. Characteristics of SARS-CoV-2 and COVID-19. Nat Rev Microbiol. 2021;19:141–54. 10.1038/s41579-020-00459-7 33024307 PMC7537588

[11] DeFraites RF. The Armed Forces Health Surveillance Center: enhancing the military health system’s public health capabilities. BMC Public Health. 2011;11(Suppl 2):S1. 10.1186/1471-2458-11-S2-S1 21388560 PMC3092410

[12] Maljkovic Berry I, Melendrez MC, Bishop-Lilly KA, Rutvisuttinunt W, Pollett S, Talundzic E, et al. Next generation sequencing and bioinformatics methodologies for infectious disease research and public health: approaches, applications, and considerations for development of laboratory capacity. J Infect Dis. 2020;221(Suppl 3):S292–307. 31612214 10.1093/infdis/jiz286

[13] Vesely BA, Cheng Q. Force protection risks in AFRICOM, INDOPACOM and SOUTHCOM due to rapid diagnostic test failures for *Falciparum* malaria, 2016–2022. MSMR. 2023;30:7–11. 37963222

[14] Kimita G, Nyataya J, Omuseni E, Sigei F, Lemtudo A, Muthanje E, et al. Temporal lineage replacements and dominance of imported variants of concern during the COVID-19 pandemic in Kenya. Commun Med (Lond). 2022;2:103. 10.1038/s43856-022-00167-8 35982756 PMC9382597

[15] Centers for Disease Control and Prevention. About One Health [cited 2024 Jan 10]. https://www.cdc.gov/onehealth/index.html

[16] Morton L, Creppage K, Rahman N, Early J, Hartman L, Hydrick A, et al. Challenges and opportunities in pathogen agnostic sequencing for public health surveillance: lessons learned from the Global Emerging Infections Surveillance Program. Health Secur. 2024;22:16–24. 10.1089/hs.2023.0068 38054950 PMC10902267

[17] Kirby AE, Walters MS, Jennings WC, Fugitt R, LaCross N, Mattioli M, et al. Using wastewater surveillance data to support the COVID-19 response—United States, 2020–2021. MMWR Morb Mortal Wkly Rep. 2021;70:1242–4. 10.15585/mmwr.mm7036a2 34499630 PMC8437053

[18] US Department of Defense. 2023 biodefense posture review [cited 2024 Jan 10]. https://media.defense.gov/2023/Aug/17/2003282337/-1/-1/1/2023_BIODEFENSE_POSTURE_REVIEW.PDF

